# Mental well‐being in prostate cancer: A multi‐institutional prospective cohort study

**DOI:** 10.1002/bco2.70040

**Published:** 2025-06-17

**Authors:** Oliver Brunckhorst, Jaroslaw Liszka, Callum James, Jack B. Fanshawe, Mohamed Hammadeh, Robert Thomas, Shahid Khan, Matin Sheriff, Gordon Muir, Hashim U. Ahmed, Mieke Van Hemelrijck, Robert Stewart, Prokar Dasgupta, Kamran Ahmed

**Affiliations:** ^1^ MRC Centre for Transplantation Guy's Hospital Campus, King's College London, King's Health Partners London UK; ^2^ Department of Psychiatry University of Oxford Oxford UK; ^3^ Department of Urology Queen Elizabeth Hospital, Lewisham and Greenwich NHS Trust London UK; ^4^ The Primrose Oncology Unit Bedfordshire Hospitals NHS Foundation Trust Bedford UK; ^5^ Department of Urology East Surrey Hospital, Surrey and Sussex Healthcare NHS Trust Redhill UK; ^6^ Department of Urology Medway NHS Foundation Trust Gillingham UK; ^7^ Department of Urology King's College Hospital NHS Foundation Trust London UK; ^8^ Imperial Prostate, Division of Surgery, Department of Surgery and Cancer Imperial College London London UK; ^9^ Imperial Urology Charring Cross Hospital, Imperial College Healthcare NHS Trust London UK; ^10^ Translational Oncology and Urology Research (TOUR), School of Cancer and Pharmaceutical Sciences King's College London London UK; ^11^ King's College London Institute of Psychiatry, Psychology and Neuroscience London UK; ^12^ South London and Maudsley NHS Foundation Trust London UK; ^13^ Department of Urology Guy's Hospital, Guy's and St Thomas' NHS Foundation Trust London UK; ^14^ Department of Urology Sheikh Khalifa Medical City Abu Dhabi United Arab Emirates; ^15^ Khalifa University Abu Dhabi United Arab Emirates

**Keywords:** anxiety, body image, depression, masculinity, prostate cancer, quality of life, survivorship

## Abstract

**Objective:**

To investigate the patient, treatment and oncological prognostic factors for multiple mental well‐being outcomes in prostate cancer.

**Patient and Methods:**

The MIND‐P study was a multi‐institutional prospective cohort study recruiting newly diagnosed prostate cancer patients for 12 months post‐diagnosis across eight centres. Periodic data collection evaluated mental, physical and social well‐being measures incorporating five mental well‐being outcomes selected based on prior research as important measures in patients with prostate cancer. This included depression, anxiety, fear of recurrence, body image, and masculinity. Treatment, patient, and oncological prognostic factors for developing significant well‐being symptoms were evaluated along with symptom trajectories.

**Results:**

Of 300 patients recruited, 13.7% and 11.0% developed depression or anxiety symptoms, with 45.0% developing at least one significant mental well‐being symptom. Those undergoing hormone monotherapy had higher depression scores from 6 months post‐diagnosis (all *p* < 0.05), with prostatectomy patients having poorer body image and masculinity scores, when compared with surveillance patients (all *p* < 0.02). Metastatic disease at diagnosis was associated with increased depression, anxiety and fear of cancer recurrence. Patient factors for poorer mental well‐being included younger age, a previous psychiatric history, social deprivation, poorer baseline mental health symptoms and poorer baseline sexual and urinary function. Symptom trajectory analysis demonstrated the increasing symptom load in body image and masculine self‐esteem experienced post any active treatment modality, with more stable scores for other mental well‐being measures.

**Conclusion:**

A high incidence of multiple mental well‐being issues was identified post‐diagnosis, highlighting their individual importance during follow‐up. Baseline mental and functional symptoms, a previous psychiatric history and stage at diagnosis appear to be particularly important prognostic factors for the development of significant symptoms. A comprehensive initial biopsychosocial assessment incorporating these could identify high‐risk patients for improved monitoring and subsequent support.

ClinicalTrials.gov number ‐ NCT04647474.

## 1 INTRODUCTION

Prostate cancer represents a significant proportion of global cancer incidence.^1^ Combined with the high and improving survival rates, this means that growing numbers now live with and beyond their disease.^2^ We are therefore increasingly aware that living longer, and surviving disease, does not always equate to living well, with quality of life in survivorship becoming an increasingly important topic, which is reflected in the increasing number of global survivorship initiatives.^3^, ^4^

The mental well‐being impact post–prostate cancer diagnosis is being increasingly appreciated.^5^ Mental health diagnoses such as depression and anxiety are prevalent, affecting roughly 17%.^6^ Additionally, issues outside of pure mental health also appear important, including fear of cancer recurrence (FCR) or progression, having been labelled as one of the most common unmet cancer needs.^7^ Furthermore, issues surrounding body image and masculinity are important, representing constructs assessing the concept of self‐worth and the view of our body and mind and how it is perceived by others.^8^ These all appear important for prostate cancer specifically, given their frequency and their association with functional outcomes, treatment choices and even mortality.^9^, ^10^

Despite this, much of the evidence base around the quality of life in prostate cancer remains centred around the physical sequelae of disease, meaning less is understood about prognostic factors associated with poorer mental well‐being. Beyond hormone therapy, little consensus exists on other patient, oncological or treatment prognostic factors, particularly for outcomes falling outside of pure mental health, such as FCR, body image and masculinity.^11^ Additionally, where evidence does exist, this is limited to general cancer populations,^12^, ^13^ with translation of findings being unclear in the prostate cancer setting given the unique experiences of these individuals. Lastly, the limited evidence specific to prostate cancer remains largely cross‐sectional, providing a weaker evidence base for prognostic factor research.

Therefore, the primary aim of this study was to explore the association between prostate cancer patients undergoing different management options and subsequent mental well‐being outcomes in the initial cancer follow‐up stage. As secondary objectives, this study also aimed to

1. Explore patient and oncological prognostic factors associated with mental well‐being outcomes in patients with prostate cancer.
2. Define the incidence of each mental well‐being outcome within the first year of diagnosis.
3. Evaluate the trajectory of mental well‐being symptoms in this period.

## 2 PATIENTS AND METHODS

Reporting of this study was conducted following the Strengthening the Reporting of Observational Studies in Epidemiology (STROBE) Statement guidelines. The full methodology of this study has been published as a protocol paper.^14^

### 2.1 Study design, setting and participants

The Mental well‐beIng aND quality of life in Prostate cancer (MIND‐P) study was a multi‐institutional prospective cohort study following newly diagnosed prostate cancer patients for 12 months. Eight secondary and tertiary hospital sites across the UK undertook data collection between 15/01/2021 and 29/03/2023. Inclusion criteria were individuals with new diagnoses of prostate cancer who were due to undergo one of four management options: active surveillance, radical prostatectomy, radical radiotherapy or hormone monotherapy. The main exclusion criteria were those who had already initiated treatment, had concurrent cancer diagnoses or were due to undergo watchful waiting, focal therapy or chemotherapy.

### 2.2 Study procedures

Individuals were followed from diagnosis for 12 months with periodic 3‐monthly questionnaire completion containing mental, physical and social well‐being measures. Primary study outcomes were validated psychometric measures for mental well‐being including five constructs deemed to be of importance to prostate cancer patients: depressive symptoms, anxiety symptoms, FCR, body image perception and masculine self‐esteem. Selection of these outcomes was undertaken through extensive background work defining mental well‐being in prostate cancer, including multiple extensive reviews^6^, ^8^, ^15^ and qualitative patient interviews.^16^, ^17^ Selection of subsequent psychometric measures for these was based on the most utilised tools, or those with the best psychometric properties within a prostate cancer population, again defined through background reviews.^6^, ^15^, ^18^ Final utilised measures for mental, physical and social well‐being and significant symptom thresholds utilised are listed in Table S1.

### 2.3 Statistical analysis

Descriptive statistics were calculated for population and study outcomes including frequencies, cumulative incidences, mean (SD) and median values (IQR). The primary analysis included an unadjusted one‐way ANOVA of each mental well‐being symptom score between management groups at 3, 6, 9 and 12‐months post‐diagnosis with subsequent Tukey's Honest Significant Difference post hoc analysis. Additionally, overall mean symptom difference across time points was assessed via a one‐way MANOVA analysis using Wilks' Lambda with a subsequent multivariate contrasts analysis. The study sample size was based on this primary analysis to meet a power of 0.8 and an alpha value of 0.05 across all five mental well‐being outcomes with an assumed dropout rate of 25%.

Additional analyses for other prognostic factors included the exploration of individual patient, oncological or treatment factors against the development of significant individual mental well‐being symptoms. An initial unadjusted, followed by an adjusted multivariate regression analysis was conducted, with adjustment factors selected based on prior background work.^11^ For mental well‐being symptom trajectory evaluation, an unadjusted and adjusted linear regression was conducted for individual mental well‐being outcomes over the study follow‐up duration.

All analyses were conducted using Stata 17.0 with a significance threshold of 0.05. Primary outcome analysis was conducted using complete case analysis with secondary analyses done after multiple imputations (20 replications) of missing data.

### 2.4 Ethics approval

Prospective ethics committee and Human Research Authority approval was gained in England (Reference: 20/LO/1136). MIND‐P was prospectively registered on ClinicalTrials.gov database (NCT04647474).

## 3 RESULTS

### 3.1 Participants

**FIGURE 1:**
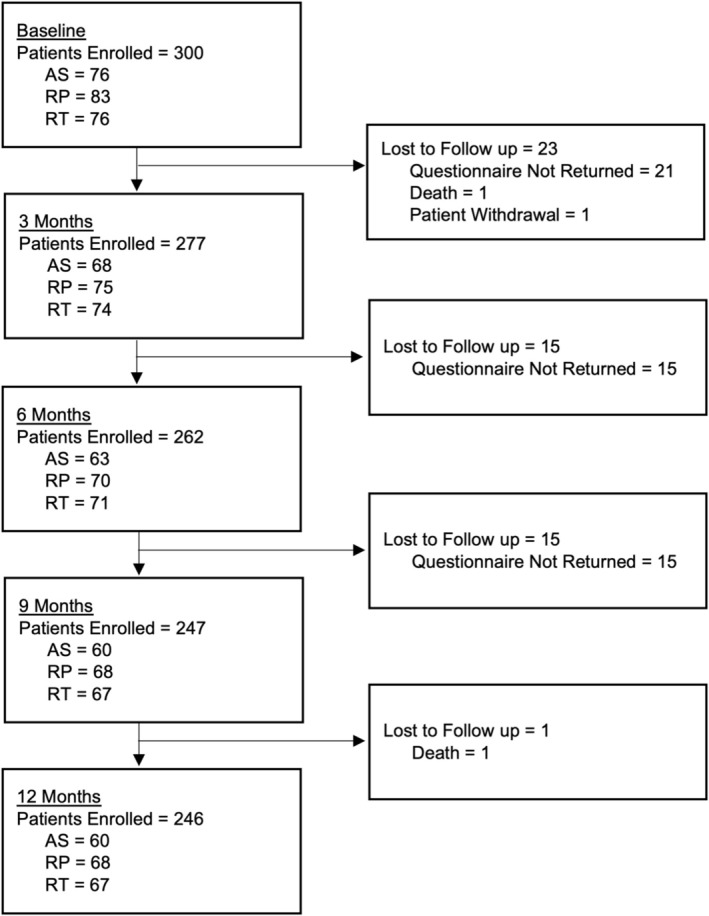
Flow diagram of study follow‐up numbers. AS, active surveillance; RP, radical prostatectomy; RT, radical radiotherapy.

Over the study duration, 300 participants were recruited, with 246 participants completing 12‐month follow‐up, accounting for an 18.0% drop‐out rate (Figure 1). Mean follow‐up was 10.3 months (SD 3.8), with a total of 257.3 person‐year follow‐up. Participant demographics are summarised in Table 1.

**TABLE 1:** Participant baseline characteristics.

Characteristic	Treatment modality				Whole cohort
	AS	RP	RT	Hormone	
*N*	76	83	76	65	300
Median age (IQR), years	67.5 (14.0)	65.0 (9.5)	70.0 (11.3)	74.0 (10.0)	68.0 (11.0)
Median PSA (IQR), μg/L	5.7 (2.8)	6.6 (3.6)	9.1 (6.4)	25.5 (97.6)	7.4 (8.8)
ISUP grade group *n* (%)					
Not available	0 (0)	0 (0)	0 (0)	3 (4.6)	3 (1.0)
1	60 (78.9)	1 (1.2)	1 (1.3)	0 (0)	62 (20.7)
2	16 (21.1)	51 (61.4)	32 (42.1)	12 (18.5)	111 (37.0)
3	0 (0)	20 (24.1)	15 (19.7)	11 (16.9)	46 (15.3)
4	0 (0)	4 (4.8)	14 (18.4)	15 (23.1)	33 (11.0)
5	0 (0)	7 (8.4)	14 (18.4)	24 (36.9)	45 (15.0)
Stage at diagnosis *n* (%)					
Localised	74 (97.4)	57 (68.7)	43 (56.6)	6 (9.2)	180 (60.0)
Locally advanced	2 (2.6)	26 (31.3)	33 (43.4)	23 (35.4)	84 (28.0)
Metastatic	0 (0)	0 (0)	0 (0)	36 (55.4)	36 (12.0)
Ethnicity *n* (%)					
White British	61 (80.3)	67 (80.7)	66 (86.8)	55 (84.6)	249 (83)
Asian or Asian British	3 (3.9)	3 (3.6)	0 (0)	2 (3.1)	8 (2.7)
Black, African, Caribbean or Black British	6 (7.9)	9 (10.8)	4 (5.3)	5 (7.7)	24 (8.0)
Any other White background	6 (7.9)	4 (4.8)	6 (7.9)	3 (4.6)	19 (6.3)
Marital status *n* (%)					
Single	7 (9.2)	9 (10.8)	7 (9.2)	8 (12.3)	31 (10.3)
Married/other cohabiting	65 (85.5)	68 (81.9)	57 (75.0)	48 (73.8)	238 (79.3)
Divorced/widowed	4 (5.3)	6 (7.2)	12 (15.8)	9 (13.8)	31 (10.3)
Employment status *n* (%)					
Employed	35 (46.1)	48 (57.8)	24 (31.6)	10 (15.4)	117 (39.0)
Unemployed/retired	41 (53.9)	35 (42.2)	52 (68.4)	55 (84.6)	183 (61.0)
Family history of PCa *n* (%)	20 (26.3)	22 (26.5)	15 (19.7)	10 (15.4)	67 (22.3)
Existing psychiatric condition *n* (%)					
Depression	4 (5.3)	0 (0)	2 (2.6)	2 (3.1)	8 (2.7)
Anxiety	1 (1.3)	1 (1.2)	1 (1.3)	2 (3.1)	5 (1.7)
Other	0 (0)	0 (0)	0	0 (0)	0 (0)
Previous psychiatric diagnosis *n* (%)	9 (11.8)	6 (7.2)	7 (9.2)	5 (7.7)	27 (9.0)
Alcohol intake *n* (%)					
Nil	19 (25.0)	13 (15.7)	18 (23.7)	16 (24.6)	66 (22.0)
Within recommended guidelines	46 (60.5)	52 (62.7)	40 (52.6)	38 (58.5)	176 (58.7)
Above recommended guidelines	11 (14.5)	18 (21.7)	18 (23.7)	11 (16.9)	58 (19.3)
Smoking status *n* (%)					
No	46 (60.5)	46 (55.4)	35 (46.1)	26 (40.0)	153 (51.0)
Yes	1 (1.3)	3 (3.6)	5 (6.6)	6 (9.2)	15 (5.0)
Ex‐smoker	29 (38.2)	34 (41)	36 (47.4)	33 (50.8)	132 (44.0)
Median index of multiple deprivation decile (IQR)	7.0 (4.3)	7.0 (5.0)	7.0 (5.0)	6.0 (4.0)	7.0 (5.0)

Abbreviations: AS, active surveillance; PCa, prostate cancer; RP, radical prostatectomy; RT, radical radiotherapy.

### 3.2 Incidence of significant symptoms

In the first year after diagnosis, the incidence of new significant depression and anxiety symptoms was 13.7% and 11.0%, respectively, excluding those with existing mental health diagnoses. Additionally, 8.0% of participants displayed suicidal ideation symptoms during this time. This is considerably less than the 5.0% who stated that they had started a new psychiatric treatment during this period. Considering other mental well‐being outcomes, 16.3% and 11.3% had significant body image and masculine self‐esteem symptoms, respectively. FCR was the most common significant symptom with an incidence of 39.0%. Overall, nearly half of individuals (45.0%) developed at least one significant mental well‐being symptom.

### 3.3 Well‐being across treatment cohorts

Primary unadjusted mean difference analysis between management groups demonstrated that those undergoing hormone monotherapy had significantly higher depressive symptoms from 6 months post‐diagnosis compared with prostatectomy patients; however, no difference was seen across other cohorts (Table 2). Those who underwent prostatectomy had significantly higher body image scores, with a gradual deterioration seen across other treatment groups. By 12 months, active surveillance patients had significantly lower body image symptom load than all other groups. Similarly, masculine self‐esteem was seen to be significantly poorer in prostatectomy patients compared with surveillance throughout, with no significant difference seen between other treatment groups. Interestingly, no difference was seen between mean scores for both anxiety and fear of recurrence outcomes across all treatment cohorts.

**TABLE 2:** Mean mental well‐being measure scores per treatment cohort.

Outcome	AS	RP	RT	Hormone	*p*‐value^a^	Sig. post hoc analysis^b^
Depression (PHQ‐9)						
Baseline	3.13 ± 3.76	3.34 ± 3.59	2.78 ± 4.09	4.15 ± 5.39	0.27	
3 months	2.85 ± 3.34	3.08 ± 3.82	3.49 ± 4.86	4.65 ± 5.07	0.09	
6 months	3.03 ± 4.10	2.39 ± 3.38	3.86 ± 4.29	4.90 ± 5.39	0.01*	Hormone vs. RP
9 months	2.70 ± 3.25	2.34 ± 2.87	3.25 ± 3.94	4.37 ± 4.13	0.02*	Hormone vs. RP
12 months	2.45 ± 3.24	2.25 ± 3.20	3.03 ± 3.88	4.14 ± 4.96	0.04*	Hormone vs. RP
Anxiety (GAD7)						
Baseline	2.78 ± 3.96	2.92 ± 3.26	2.17 ± 3.52	3.35 ± 4.83	0.34	
3 months	2.85 ± 3.42	2.48 ± 3.91	2.38 ± 3.88	3.6 ± 5.04	0.31	
6 months	2.62 ± 3.85	2.01 ± 3.31	2.41 ± 3.03	3.66 ± 4.40	0.08	
9 months	2.47 ± 3.47	1.96 ± 2.84	1.69 ± 2.88	3.11 ± 4.25	0.11	
12 months	2.33 ± 2.94	2.04 ± 3.12	2.10 ± 3.36	3.14 ± 4.18	0.31	
Body image (BIS)						
Baseline	2.27 ± 3.19	2.76 ± 3.73	2.09 ± 2.62	2.64 ± 4.68	0.62	
3 months	1.75 ± 2.62	5.07 ± 5.15	3.08 ± 3.88	2.97 ± 4.28	<0.001*	RP vs All
6 months	2.22 ± 3.99	4.58 ± 4.30	3.62 ± 4.32	3.50 ± 4.36	0.02*	RP vs. AS
9 months	1.93 ± 3.37	4.49 ± 4.24	4.10 ± 5.23	3.19 ± 3.53	<0.01*	RP and RT vs AS
12 months	1.45 ± 2.28	4.75 ± 4.95	4.78 ± 6.12	4.08 ± 4.60	<0.001*	All vs. AS
Fear of recurrence (FCR7)						
Baseline	12.31 ± 5.86	14.51 ± 6.58	13.05 ± 6.01	13.47 ± 7.25	0.2	
3 months	12.84 ± 6.92	14.28 ± 6.87	13.11 ± 6.58	14.49 ± 8.34	0.44	
6 months	13.75 ± 7.45	12.81 ± 6.46	12.01 ± 4.97	14.70 ± 7.96	0.13	
9 months	13.95 ± 8.47	12.67 ± 5.91	11.58 ± 5.66	14.45 ± 7.37	0.1	
12 months	13.65 ± 6.91	12.57 ± 5.44	12.42 ± 5.30	14.51 ± 8.32	0.26	
Masculine self‐esteem (PC‐QoL)						
Baseline	92.46 ± 11.80	87.82 ± 15.52	90.71 ± 12.33	91.46 ± 13.34	0.16	
3 months	93.74 ± 11.65	84.17 ± 17.89	88.47 ± 16.68	88.91 ± 15.64	<0.01*	RP vs. AS
6 months	93.05 ± 14.74	83.55 ± 17.61	87.41 ± 14.68	87.05 ± 16.42	<0.01*	RP vs. AS
9 months	93.06 ± 13.51	82.22 ± 19.19	87.59 ± 15.73	89.06 ± 13.44	<0.01*	RP vs. AS
12 months	92.94 ± 14.54	83.23 ± 18.08	85.10 ± 20.13	87.13 ± 18.38	0.02*	RP vs. AS

Abbreviations: AS, active surveillance; BIS, body image scale; FCR7, Fear of Cancer Recurrence 7 Scale; GAD7, Generalised Anxiety Disorder Assessment 7; PC‐QoL, Prostate Cancer Quality of Life Masculine Self‐Esteem Subscale; PHQ‐9, Patient Health Questionnaire 9; RP, radical prostatectomy; RT, radical radiotherapy.

^a^ One‐way ANOVA.

^b^ Tukey's Honest Significant Difference post hoc test.

* Statistically significant.

Evaluating overall mean differences of symptom scores on MANOVA across follow‐up revealed similar results (Table S2). Mean depressive scores were higher for those undergoing hormone treatments and radiotherapy (both *p* = 0.03) compared with prostatectomy. For body image perception, those undergoing surveillance displayed better symptom scores compared with all other groups (all *p* < 0.001), with those undergoing prostatectomy having poorer scores compared with all others (all *p* < 0.001). Similarly, for masculinity, prostatectomy patients had poorer scores compared with surveillance participants (*p* = 0.04). No differences were seen in anxiety scores. Lastly, surveillance patients displayed higher FCR scores when compared with prostatectomy patients (*p* = 0.03).

### 3.4 Mental well‐being prognostic factors

Table S3 summarises the unadjusted analysis exploring patient, oncological and treatment factors for individual well‐being outcomes with adjusted analysis demonstrated in Table 3. Post‐adjustment, increasing age was a significant protective factor for the development of body image and masculine self‐esteem issues (aOR, 0.95) but was not related to depression, anxiety or FCR. Increased social deprivation was associated with poorer depression, anxiety and masculinity outcomes (aOR, 0.82–0.86). Although Black ethnicity was related to poorer depressive symptoms (aOR, 3.14), ethnicity was not associated with other outcomes. A previous psychiatric history was a strong predictor for poorer outcomes across all domains (aOR, 2.97–3.89). There was however little association between marital or employment status, family history, alcohol intake, smoking and comorbidities.

**TABLE 3:** Adjusted analysis of prognostic factors for mental well‐being outcomes in the first year of diagnosis.

Prognostic factor	Depression	Anxiety	Body image	Fear of cancer recurrence	Masculine self‐esteem
	aOR	aOR	aOR	aOR	aOR
	(95% CI, *p*‐value)	(95% CI, *p*‐value)	(95% CI, *p*‐value)	(95% CI, *p*‐value)	(95% CI, *p*‐value)
Patient factors					
Age	0.99 (0.95–1.04, *p* = 0.81)	0.99 (0.94–1.04, *p* = 0.73)	**0.95 (0.91–0.99, *p* = 0.02)** *	0.97 (0.94–1.01, *p* = 0.12)	**0.95 (0.91–1.00, *p* = 0.04)** *
Ethnicity					
White British	Ref	Ref	Ref	Ref	Ref
Asian or Asian British	1.02 (0.12–8.93, *p* = 0.98)	1.30 (0.15–11.62, *p* = 0.81)	‐	0.52 (0.10–2.68, *p* = 0.44)	‐
Black, African, Caribbean or Black British	**3.19 (1.04–9.77, *p* = 0.04)** *	1.87 (0.50–6.99, *p* = 0.35)	1.08 (0.30–3.90, *p* = 0.91)	2.15 (0.78–5.94, *p* = 0.14)	0.72 (0.14–3.78, *p* = 0.70)
Any other White background	‐	‐	0.64 (0.13–3.03, *p* = 0.57)	1.24 (0.45–3.37, *p* = 0.68)	1.48 (0.38–5.70, *p* = 0.57)
Marital status					
Single	Ref	Ref	Ref	Ref	Ref
Married/other cohabiting	3.91 (0.48–31.80, *p* = 0.20)	3.58 (0.45–28.58, *p* = 0.23)	1.29 (0.40–4.15, *p* = 0.67)	1.03 (0.46–2.31, *p* = 0.95)	0.95 (0.30–3.08, *p* = 0.94)
Divorced/widowed	4.28 (0.42–43.78, *p* = 0.22)	3.90 (0.38–39.86, *p* = 0.25)	2.13 (0.50–9.03, *p* = 0.30)	1.01 (0.34–2.99, *p* = 0.98)	0.84 (0.16–4.47, *p* = 0.84)
Employment status					
Employed	Ref	Ref	Ref	Ref	Ref
Unemployed	‐	5.41 (0.41–20.75, *p* = 0.20)	0.89 (0.07–10.04, *p* = 0.93)	1.61 (0.13–19.23, *p* = 0.71)	1.23 (0.11–14.12, *p* = 0.87)
Retired	1.07 (0.41–2.79, *p* = 0.88)	1.40 (0.48–4.09, *p* = 0.54)	0.46 (0.19–1.09, *p* = 0.08)	0.93 (0.48–1.82, *p* = 0.83)	0.67 (0.25–1.78, *p* = 0.42)
Family history of prostate cancer	1.62 (0.73–3.61, *p* = 0.24)	1.80 (0.76–0.46, *p* = 0.18)	1.14 (0.53–2.44, *p* = 0.74)	0.97 (0.53–1.76, *p* = 0.91)	0.92 (0.37–2.26, *p* = 0.85)
Previous psychiatric diagnosis	**3.35 (1.11–10.05, *p* = 0.03)** *	**3.63 (1.21–10.84, *p* = 0.02)** *	**3.89 (1.63–9.31, *p* = 0.002)** *	**2.97 (1.26–6.99, *p* = 0.01)** *	**3.00 (1.15–7.82, *p* = 0.02)** *
Charlson comorbidity index	1.23 (0.84–1.80, *p* = 0.29)	1.31 (0.87–1.95, *p* = 0.19)	0.93 (0.58–1.48, *p* = 0.76)	0.84 (0.61–1.15, *p* = 0.27)	1.10 (0.68–1.80, *p* = 0.68)
Alcohol intake					
Nil	Ref	Ref	Ref	Ref	Ref
Within recommended guidelines	**0.37 (0.16–0.85, *p* = 0.02)** *	1.00 (0.37–2.68, *p* = 0.99)	1.10 (0.45–2.69, *p* = 0.84)	0.78 (0.41–1.51, *p* = 0.47)	1.25 (0.46–3.38, *p* = 0.67)
Above recommended guidelines	**0.27 (0.09–0.84, *p* = 0.02)** *	0.71 (0.20–2.47, *p* = 0.59)	1.01 (0.36–2.89, *p* = 0.98)	0.87 (0.40–1.93, *p* = 0.74)	0.57 (0.15–2.18, *p* = 0.41)
Smoking status					
No	Ref	Ref	Ref	Ref	Ref
Yes	1.65 (0.42–6.44, *p* = 0.47)	1.91 (0.44–8.32, *p* = 0.39)	1.28 (0.33–4.88, *p* = 0.72)	0.93 (0.28–3.10, *p* = 0.90)	1.17 (0.28–4.81, *p* = 0.83)
Ex‐smoker	0.52 (0.23–1.14, *p* = 0.10)	1.09 (0.49–2.47, *p* = 0.83)	0.81 (0.41–1.60, *p* = 0.54)	0.84 (0.51–1.40, *p* = 0.51)	**0.37 (0.15–0.87, *p* = 0.02)** *
Indices of multiple deprivation decile	**0.84 (0.072–0.97, *p* = 0.02)** *	**0.82 (0.69–0.96, *p* = 0.02)** *	0.95 (0.84–1.08, *p* = 0.45)	0.92 (0.83–1.02, *p* = 0.13)	**0.86 (0.74–0.99, *p* = 0.04)** *
Baseline mental health					
Depressive symptoms	**1.38 (1.24–1.54, *p* < 0.001)** *	**1.26 (1.15–1.37, *p* < 0.001)** *	**1.19 (1.10–1.28, *p* < 0.001)** *	**1.18 (1.09–1.27, *p* < 0.001)** *	**1.20 (1.11–1.30, *p* < 0.001)** *
Anxiety symptoms	**1.36 (1.21–1.52, *p* < 0.001)** *	**1.40 (1.23–1.58, *p* < 0.001)** *	**1.22 (1.12–1.33, *P* < 0.001)** *	**1.31 (1.19–1.46, *p* < 0.001)** *	**1.27 (1.15–1.40, *p* < 0.001)** *
Baseline EPIC‐26 score					
Urinary incontinence	**0.98 (0.96–0.99, *p* = 0.003)** *	0.99 (0.97–1.00, *p* = 0.15)	**0.99 (0.97–1.00, *p* = 0.003)** *	0.99 (0.98–1.00, *p* = 0.09)	**0.98 (0.97–1.00, *p* = 0.02)** *
Urinary irritative/obstructive	**0.97 (0.95–0.99, *p* = 0.01)** *	0.98 (0.96–1.01, *p* = 0.12)	**0.96 (0.94–0.98, *p* < 0.001)** *	**0.97 (0.95–0.98, *p* < 0.001)** *	**0.96 (0.94–0.99, *p* = 0.001)** *
Sexual	**0.98 (0.96–0.99, *p* = 0.002)** *	**0.98 (0.96–0.99, *p* = 0.01)** *	**0.98 (0.97–0.99, *p* = 0.002)** *	**0.99 (0.98–1.00, *p* = 0.01)** *	**0.98 (0.96–0.99, *p* = 0.002)** *
Baseline social well‐being score	0.95 (0.89–1.01, *p* = 0.08)	0.90 (0.84–0.96, *p* = 0.002)*	0.95 (0.90–1.01, *p* = 0.12)	0.94 (0.90–0.98, *p* = 0.01)*	0.94 (0.88–1.00, *p* = 0.05)
Short form 12 physical component score	**0.92 (0.89–0.96, *p* < 0.001)** *	0.98 (0.94–1.02, *p* = 0.26)	0.97 (0.94–1.01, *p* = 0.15)	**0.97 (0.94–1.00, *p* = 0.03)** *	**0.95 (0.91–0.99, *p* = 0.01)** *
Oncological factors					
PSA at diagnosis	1.00 (1.00–1.00, *p* = 0.98)	1.00 (1.00–1.00, *p* = 0.79)	1.00 (1.00–1.00, *p* = 0.78)	1.00 (1.00–1.00, *p* = 0.28)	1.00 (1.00–1.00, *p* = 0.58)
ISUP grade	1.06 (0.81–1.39, *p* = 0.67)	1.25 (0.93–1.68, *p* = 0.13)	**1.45 (1.13–1.87, *p* = 0.004)** *	1.11 (0.91–1.34, *p* = 0.30)	**1.49 (1.12–1.98, *p* = 0.01)** *
Stage					
Localised	Ref	Ref	Ref	Ref	Ref
Locally advanced	**2.39 (1.05–5.43, *p* = 0.04)** *	1.93 (0.79–4.73, *p* = 0.15)	**3.46 (1.68–7.10, *p* = 0.001)** *	1.19 (0.67–2.09, *p* = 0.55)	**3.44 (1.53–7.72, *p* = 0.003)** *
Metastatic	**4.09 (1.44–11.63, *p* = 0.01)** *	**3.58 (1.16–11.03, *p* = 0.03)** *	1.51 (0.44–5.18, *p* = 0.51)	**2.94 (1.30–6.64, *p* = 0.01)** *	1.93 (0.48–7.70, *p* = 0.35)
Treatment factors					
Treatment modality					
Active surveillance	Ref	Ref	Ref	Ref	Ref
Prostatectomy	0.63 (0.21–1.89, *p* = 0.41)	1.51 (0.43–5.28, *p* = 0.52)	**4.17 (1.33–13.05, *p* = 0.01)** *	0.68 (0.33–1.39, *p* = 0.29)	3.15 (0.94–10.49, *p* = 0.06)
Radiotherapy	1.12 (0.39–3.23, *p* = 0.84)	1.89 (0.51–7.04, *p* = 0.34)	**3.47 (1.09–11.08, *p* = 0.04)** *	0.71 (0.35–1.45, *p* = 0.35)	2.64 (0.74–9.43, *p* = 0.14)
Hormone monotherapy	2.65 (0.93–7.60, *p* = 0.07)	**5.06 (1.30–18.25, *p* = 0.01)** *	**3.88 (1.10–13.71, *p* = 0.04)** *	1.60 (0.75–3.42, *p* = 0.22)	3.87 (0.99–15.02, *p* = 0.05)

*Note*: Adjusted for age, ethnicity, marital status, comorbidities and previous psychiatric history.

Abbreviations: EPIC‐26, Expanded Prostate Cancer Index Composite Short Form; ISUP, International Society of Urological Pathology; PSA, prostate‐specific antigen.

* Statistically significant.

Baseline well‐being status appeared important. Baseline depressive and anxiety scores were strongly associated with developing any significant mental well‐being issue (aOR, 1.18–1.40). Similarly, baseline urinary and particularly sexual function scores were predictive of subsequent significant mental well‐being symptoms across most outcomes. Lastly, baseline poorer social well‐being scores appeared to be associated with anxiety and FCR, with baseline physical function associated with depression, FCR and masculine self‐esteem.

Oncological factors had a less certain association with all outcomes. PSA values demonstrated no relationship with any outcome, with increasing ISUP grade only associated with poorer body image and masculine self‐esteem. However, those with localised disease, in general, had better outcomes than those with locally advanced or metastatic disease at presentation.

### 3.5 Symptom trajectories

**FIGURE 2:**
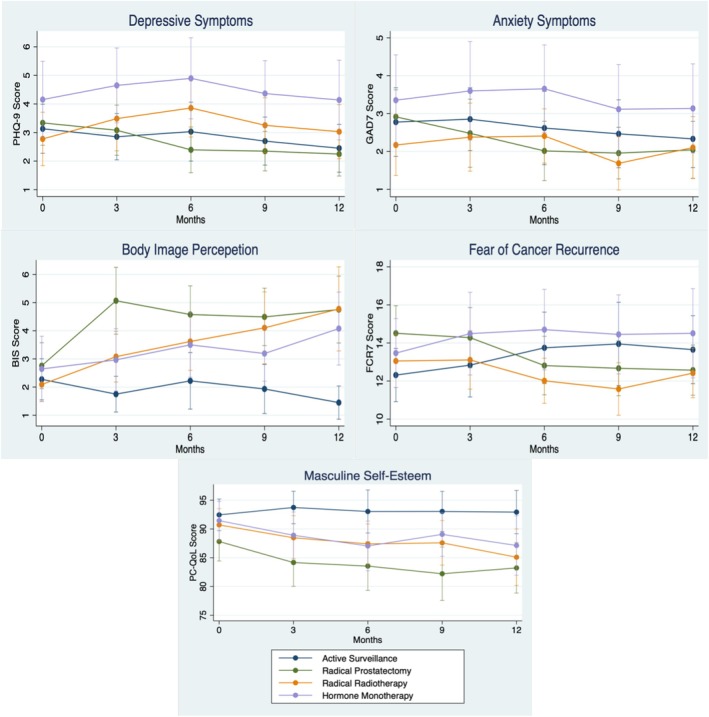
Trajectory of mean mental well‐being outcome scores divided by management modality undergone.

Overall, both unadjusted and adjusted linear regressions showed mildly reducing anxiety symptoms over the first year of diagnosis (Table S4). Conversely, body image and masculinity symptoms increased moderately in time. Lastly, there appeared to be little relationship between depressive and fear of recurrence symptom scores and time since diagnosis. Figure 2 demonstrates the mean symptom score trajectories per treatment cohort, demonstrating that although depressive symptoms appeared to increase initially for those undergoing prostatectomy and hormone monotherapy, these appeared to reduce back to baseline levels by 12 months. Similarly, anxiety symptoms appeared relatively static except when considering those who underwent prostatectomy who appeared to have a gradual decline in symptom scores, a trend also seen for FCR. Lastly, as expected, symptoms of body image disturbance and masculine self‐esteem deteriorated with time in those who had undergone any form of active treatment.

## 4 DISCUSSION

The MIND‐P study aimed to better understand the mental well‐being impact post–prostate cancer diagnosis, with a focus on exploring prognostic factors for multiple outcomes. We demonstrate the significant mental well‐being morbidity that exists in this population early post‐diagnosis. The initial 1–3 years has previously been highlighted as a key monitoring period for mental health issues, with the significant incidence of new symptoms seen across all outcomes in our cohort a good demonstration of why this is the case.^19^

Evaluating the role of treatment on mental well‐being demonstrated that although depressive symptoms were greater in hormone patients, there was interestingly little difference in anxiety scores between treatment groups. However, those undergoing prostatectomy had significantly higher masculine self‐esteem issues than those who underwent surveillance, with body image issues increasingly more common in those undergoing any form of active treatment. Lastly, the role of treatment on FCR was less clear, with only extended MANOVA analysis demonstrating poorer symptoms in those undergoing surveillance. These findings were supported by symptom trajectory analysis, which demonstrated the increasing symptom load in body image and masculine self‐esteem experienced post treatment. This is largely expected considering the effect treatments can have on functional outcomes and the relationship these have with both body image and masculinity.^8^, ^20^, ^21^, ^22^

When evaluating other patient and oncological prognostic factors for mental well‐being, some demonstrated a consistent relationship with multiple outcomes. A previous psychiatric diagnosis along with baseline mental health, social well‐being, physical and functional symptoms scores were all strongly associated with poorer mental well‐being. However, other factors, particularly age, social deprivation and stage of disease at presentation, had less certain associations because of their significance being seen across more selected outcomes. Lastly, this study did identify several factors that appeared to have no relationship with subsequent mental well‐being, with comorbidities, marital status, smoking status, alcohol intake and PSA at diagnosis as important examples of this.

These findings appear to be consistent with the limited previous evidence available, particularly around the role of previous psychiatric history and baseline mental/physical status.^11^ However, as previously highlighted, limited prior longitudinal evidence existed for outcomes outside of pure mental health. This study therefore adds significantly to this evidence base, with findings that could focus on future confirmatory prognostic factor research. This could arise through larger scale dedicated individual studies, through the inclusion of mental well‐being outcomes within other longitudinal studies, or through the increasing collaborative big data platforms being undertaken.

Our findings have important clinical implications. A high incidence of mental well‐being issues was seen, with findings that 45% of individuals suffered at least one significant symptom serving as a stark reminder of the importance of these outcomes. This supports existing survivorship recommendations surrounding the need to screen for the psychosocial effects of prostate cancer during care.^23^, ^24^ This is important given the multiple interventions and prevention strategies such as cognitive therapy, mindfulness‐based therapy, exercise and technology interventions, which have been highlighted as effective measures to improve these outcomes.^25^, ^26^, ^27^, ^28^ Additionally, post‐prostatectomy issues surrounding masculinity and body image appeared to be more severe, highlighting one important group requiring targeted attention. Survivorship care post‐prostatectomy remains unstructured and should consider these issues with several options having been demonstrated to be effective in this group specifically including exercise or targeted behavioural therapy.^27^, ^29^ Lastly, these results highlight the importance of a comprehensive biopsychosocial assessment at baseline. The strong prognostic role of baseline mental health, social well‐being and functional symptoms demonstrates this and could highlight patients at particular risk.

It is important to consider the limitations of the MIND‐P study. Firstly, the 12‐month study follow‐up remains relatively short. Although the initial period is a key time for psychosocial care, within the context of the long average prostate cancer survival, this remains an important consideration.^19^ Secondly, the results should be taken within the context of the COVID‐19 pandemic, which has been demonstrated to have had an impact on mental health prevalences and limited concurrent partner recruitment.^30^ Lastly, it is important to consider the study population itself. Findings are ultimately limited to the four recruited treatment groups with those undergoing chemotherapy or focal therapies excluded during recruitment. The wide confidence intervals surrounding ethnicity evaluations due to limited events in these cohorts also highlight the limited conclusions that may be drawn from our analysis on this, meaning that future evaluation of these factors is important to ensure that the evidence reflects the populations encountered in clinical practice.

## 5 CONCLUSION

We highlight the importance of considering mental well‐being issues post–prostate cancer diagnosis given their significant incidences. Additionally, potential prognostic factors for poorer outcomes included the role of hormone therapy in depression and undergoing prostatectomy for body image and masculine self‐esteem. Moreover, a previous psychiatric diagnosis, stage at diagnosis, age, social deprivation and baseline mental, functional and social status may all act as potentially important patient and oncological factors. These highlight potential factors to consider clinically, with a comprehensive biopsychosocial assessment at baseline offering one way to identify higher risk patients.

## AUTHOR CONTRIBUTIONS

Conception and design: Oliver Brunckhorst, Hashim U. Ahmed, Mieke Van Hemelrijck, Robert Stewart, Prokar Dasgupta, and Kamran Ahmed. Acquisition of data: Oliver Brunckhorst, Jaroslaw Liszka, Callum James, Jack B. Fanshawe, Mohamed Hammadeh, Robert Stewart, Shahid Khan, Matin Sheriff, Gordon Muir, Hashim U. Ahmed, Prokar Dasgupta, and Kamran Ahmed. Analysis and interpretation of data: Oliver Brunckhorst, Mieke Van Hemelrijck, Prokar Dasgupta, and Kamran Ahmed. Drafting of the manuscript: Oliver Brunckhorst. Critical revision of the manuscript for important intellectual content: Jaroslaw Liszka, Callum James, Jack B. Fanshawe, Mohamed Hammadeh, Robert Thomas, Shahid Khan, Matin Sheriff, Gordon Muir, Hashim U. Ahmed, Mieke Van Hemelrijck, Robert Stewart, Prokar Dasgupta, and Kamran Ahmed. Statistical analysis: Oliver Brunckhorst, Mieke Van Hemelrijck, Prokar Dasgupta, and Kamran Ahmed. Obtaining funding: Oliver Brunckhorst, Prokar Dasgupta, and Kamran Ahmed. Supervision: Robert Stewart, Prokar Dasgupta, and Kamran Ahmed.

## CONFLICT OF INTEREST STATEMENT

P.D. is advisor to Proximie and Chief Medical Officer to MysteryVibe.

## Supporting information


**Table S1** Definitions used for threshold of significance across mental well‐being constructs.
**Table S2** Mean mental well‐being analysis per treatment cohort across study follow‐up.
**Table S3** Unadjusted analysis of prognostic factors for mental well‐being outcomes in the first year of diagnosis.
**Table S4** Relationship between time since diagnosis and individual mental well‐being outcomes.

## Data Availability

The data that support the findings of this study are available on request from the corresponding author. The data are not publicly available due to privacy or ethical restrictions.
