# Supplementary material for: Mental well‐being in prostate cancer: A multi‐institutional prospective cohort study
Source: BJUI Compass. 2025 Jun 17;6(6):e70040. doi: 10.1002/bco2.70040 (PMC12171242; doi:10.1002/bco2.70040)
Supplement: Supplementary file 1 — Table S1 Definitions used for threshold of significance across mental well‐being constructs. Table S2 Mean mental well‐being analysis per treatment cohort across study follow‐up. Table S3 Unadjusted analysis of prognostic factors for mental well‐being outcomes in the first year of diagnosis. Table S4 Relationship between time since diagnosis and individual mental well‐being outcomes. [file BCO2-6-e70040-s001.docx]

**Supplementary Materials**

| Mental Wellbeing Construct | Psychometric Tool | Threshold for Significance |
| --- | --- | --- |
| Depression | PHQ-9 | ≥ 10 |
| Anxiety | GAD-7 | ≥ 10 |
| Fear of Cancer Recurrence/Progression | FCR7 | ≥ 17 |
| Body Image Perception | BIS | ≥ 10 |
| Masculinity | PC-QoL | < 60 |
| *Index: BIS – Body Image Scale, FCR7 – Fear of Recurrence Scale 7, GAD-7 - Generalised Anxiety Disorder – 7, PC-QoL - Prostate Cancer-Quality of Life (PC-QoL) Masculine Self-Esteem Subset, PHQ-9 - Patient Health Questionnaire – 9* | | |

**Supplementary Table 1** – Definitions used for threshold of significance across mental wellbeing constructs

| **Outcome** | **P Value †** | **Sig. Post-Hoc Analysis §** | **Direction of Effect** |
| --- | --- | --- | --- |
| **Depression (PHQ-9)** | **0.03*** | RP vs. RT and Hormone | RT and Hormone increased depression |
| **Anxiety (GAD7)** | 0.24 | N/A | N/A |
| **Body Image (BIS)** | **<0.001*** | AS vs. All  RP vs. All | AS decreased body image issues  RP increased body image issues |
| **Fear of Recurrence (FCR7)** | **0.03*** | AS vs. RP | AS increased fear of recurrence |
| **Masculine Self-Esteem (PC-QoL)** | **0.04*** | AS vs. RP | RP increased masculinity issues |
| *Index: AS – Active Surveillance, BIS – Body Image Scale, FCR7 – Fear of Cancer Recurrence 7 Scale, GAD7 – Generalised Anxiety Disorder Assessment – 7, PC-QoL - Prostate Cancer Quality of Life Masculine Self-Esteem Subscale, PHQ-9 - Patient Health Questionnaire – 9, RP – Radical Prostatectomy, RT – Radical Radiotherapy*  **** Statistically Significant***  *† One-way MANOVA using Wilks’ Lambda*  *§ Multivariate contrasts analysis. post hoc test* | | | |

**Supplementary Table 2** – Mean mental wellbeing analysis per treatment cohort across study follow-up.

| **Prognostic Factor** | **Depression**  **OR**  **(95% CI, p-value)** | **Anxiety**  **OR**  **(95% CI, p-value)** | **Body Image**  **OR**  **(95% CI, p-value)** | **Fear of Cancer Recurrence**  **OR**  **(95% CI, p-value)** | **Masculine Self-Esteem**  **OR**  **(95% CI, p-value)** |
| --- | --- | --- | --- | --- | --- |
| *Patient Factors* | | | | | |
| Age | 0.98 (0.94-1.02, p=0.35) | 0.98 (0.94-1.03, p=0.46) | **0.95 (0.91-0.98, p=0.01)*** | **0.96 (0.94-0.99, p=0.02)*** | **0.95 (0.91-0.99, p=0.03)*** |
| Ethnicity  White British  Asian or Asian British  Black, African, Caribbean, or Black British  Any other White Background | Ref  0.90 (0.11-7.57, p=0.92)  **3.16 (1.14-8.77, p=0.03)***  - | Ref  1.13 (0.11-7.57, p=0.92)  1.95 (0.59-6.45, p=0.27)  -- | Ref  -  1.15 (0.34-3.87, p=0.82)  0.53 (0.12-2.39, p=0.41) | Ref  0.49 (0.10-2.46, p=0.34)  2.05 (0.80-5.25, p=0.13)  1.16 (0.53-3.05, p=0.77) | Ref  -  0.90 (0.19-24.40, p=0.90)  1.59 (0.41-6.12 p=0.50) |
| Marital Status  Single  Married/Other Co-Habiting  Divorced/ Widowed | Ref  4.24 (0.56-32.26, p=0.16)  4.64 (0.50-42.75, p=0.18) | Ref  3.65 (0.48-27.96, p=0.21)  4.09 (0.43-39.18, p=0.22) | Ref  1.27 (0.41-3.86, p=0.68)  1.79 (0.46-6.95, p=0.40) | Ref  0.99 (0.45-2.15, p=0.97)  0.90 (0.32-2.54, p=0.84) | Ref  0.86 (0.28-2.67, p=0.80)  0.67 (0.14-3.35, p=0.63) |
| Employment Status  Employed  Unemployed  Retired | Ref  11.36(0.97-90.05, p=0.05)  0.86 (0.43-1.72, p=0.68) | Ref  2.72 (0.26-28.50, p=0.40)  1.02 (0.47-2.22, p=0.96) | Ref  0.98 (0.10-10.01, p=0.99)  **0.37 (0.20-0.70, p=0.002)*** | Ref  1.71 (0.16-18.75, p=0.66)  0.67 (0.41-1.09, p=0.11) | Ref  1.61 (0.16-16.33, p=0.69)  **0.47 (0.23-0.96, p=0.04)*** |
| Family History of Prostate Cancer | 1.70 (0.82-3.56, p=0.16) | 1.78 (0.80-3/98, p=0.16) | 1.25 (0.61-2.56, p=0.54) | 1.11 (0.63-1.96, p=0.72) | 0.95 (0.40-2.24, p=0.91) |
| Previous Psychiatric Diagnosis | **3.02 (1.07-8.51, p=0.04)*** | **3.00 (1.09-8.27, p=0.03)*** | **4.52 (1.96-10.47, p<0.001)*** | **3.07 (1.33-7.11, p=0.01)*** | **3.55 (1.41-8.92, p=0.01)*** |
| Charlson Comorbidity Index | 1.27 (0.89-1.78, p=0.20) | 1.26 (0.87-1.84, p=0.22) | 0.82 (0.52-1.28, p=0.39) | **0.90 (0.82-0.99, p=0.03)*** | 0.96 (0.61-1.51, p=0.86) |
| Alcohol Intake  Nil  Within Recommended Guidelines  Above Recommended Guidelines | Ref  **0.32 (0.15-0.69,p=0.004)***  **0.28 (0.10-0.82, p=0.02)*** | Ref  0.81 (0.33-1.98, p=0.65)  0.67 (0.20-2.16, p=0.50) | Ref  1.19 (0.52-2.73, p=0.68)  1.20 (0.45-3.21, p=0.71) | Ref  0.78 (0.42-1.44, p=0.43)  0.92 (0.44-1.95, p=0.84) | Ref  1.44 (0.56-3.68, p=0.45)  0.71 (0.19-2.57, p=0.60) |
| Smoking Status  No  Yes  Ex-Smoker | Ref  2.08 (0.59-7.31, p=0.25)  0.50 (0.24-1.05, p=0.07) | Ref  2.42 (0.61-9.69, p=0.21)  1.00 (0.46-2.19, p=0.99) | Ref  1.75 (0.51-6.02, p=0.37)  0.73 (0.39-1.39, p=0.34) | Ref  1.09 (0.35-3.39, p=0.89)  0.77 (0.47-1.25, p=0.29) | Ref  1.39 (0.36-5.35, p=0.63)  **0.38 (0.17-0.86, p=0.02)*** |
| Indices of Multiple Deprivation Decile | **0.85 (0.74-0.97, p=0.01)*** | **0.85 (0.73-0.98, p=0.02)*** | 0.94 (0.84-1.06, p=0.30) | **0.85 (0.73-0.98, p=0.02)*** | **0.86 (0.75-0.99, p=0.03)*** |
| Baseline Mental Health  Depressive Symptoms  Anxiety Symptoms | **1.34 (1.22-1.47,p<0.001)***  **1.33 (1.21-1.47,P<0.001)*** | **1.24 (1.14-1.34, p<0.001)***  **1.33 (1.20-1.47, P<0.001)*** | **1.18 (1.10-1.27, p<0.001)***  **1.23 (1.13-1.33, P<0.001)*** | **1.19 (1.11-1.28, p<0.001)***  **1.33 (1.20-1.46, p<0.001)*** | **1.21 (1.12-1.30, p<0.001)***  **1.27 (1.17-1.39, p<0.001)*** |
| Baseline EPIC-26 Score  Urinary Incontinence  Urinary Irritative/Obstructive  Sexual | **0.98 (0.97-0.99,p=0.004)***  **0.97 (0.96-0.99, p=0.01)***  **0.98 (0.97-0.99,p=0.006)*** | 0.99 (0.97-1.01, p=0.22)  0.98 (0.96-1.01, p=0.16)  **0.98 (0.97-1.00, p=0.02)*** | 0.99 (0.98-1.00, p=0.09)  **0.97 (0.95-0.99, p=0.001)***  0.99 (0.98-1.00, p=0.08) | 0.99 (0.98-1.00, p=0.11)  **0.97 (0.95-0.98, p<0.001)***  0.99 (0.99-1.00, p=0.10) | **0.98 (0.97-1.00, p=0.04)***  **0.97 (0.95-0.99, p=0.002)***  **0.99 (0.97-1.00, p=0.04)*** |
| Baseline Social Wellbeing Score | 0.96 (0.91-1.01, p=0.12) | **0.92 (0.87-0.97, p=0.004)*** | 0.96 (0.91-1.01, p=0.13) | **0.94 (0.90-0.99, p=0.01)*** | 0.95 (0.89-1.01, p=0.08) |
| Short Form 12 Physical Component Score | **0.95 (0.91-0.98,p=0.001)*** | **0.95 (0.91-0.98, p=0.001)*** | 0.99 (0.96-1.02, p=0.55) | 0.98 (0.95-1.01, p=0.14) | **0.96 (0.93-1.00, p=0.04)*** |
| *Oncological Factors* | | | | | |
| PSA at Diagnosis | 1.00 (1.00-1.00, p=0.88) | 1.00 (1.00-1.00, p=0.72) | 1.00 (1.00-1.00, p=0.80) | 1.00 (1.00-1.00, p=0.49) | 1.00 (1.00-1.00, p=0.99) |
| ISUP Grade | 1.07 (0.84-1.37, p=0.58) | 1.21 (0.93-1.58, p=0.16) | **1.25 (1.00-1.56, p<0.05)*** | 1.03 (0.40-1.13, p=0.13) | 1.28 (0.99-1.66, p=0.06) |
| Stage  Localised  Locally Advanced  Metastatic | Ref  2.09 (0.97-4.50, p=0.06)  **3.12 (1.24-7.82, p=0.02)*** | Ref  1.71 (0.73-4.00, p=0.21)  **2.77 (1.01-7.57, p<0.05) *** | Ref  **2.89 (1.49-5.61, p=0.002)***  0.89 (0.28-2.80, p=0.84) | Ref  1.09 (0.64-1.86, p=0.76)  1.84 (0.88-3.87, p=0.11) | Ref  **2.94 (1.37-6.28, p=0.01)***  1.05 (0.28-3.86, p=0.95) |
| *Treatment Factors* | | | | | |
| Treatment Modality  Active Surveillance  Prostatectomy  Radiotherapy  Hormone Monotherapy | Ref  0.71 (0.25-1.99, p=0.51)  0.95 (0.36-2.54, p=0.92)  1.88 (0.76-4.67, p=0.17) | Ref  1.33 (0.41-4.32, p=0.63)  1.35 (0.41-4.50, p=0.62)  2.98 (0.99-8.94, p=0.05) | Ref  **3.80 (1.35-10.65, p=0.01)***  2.56 (-.89-7.35, p=0.08)  2.07 (0.67-6.39, p=0.20) | Ref  0.78 (0.40-1.52, p=0.46)  0.62 (0.32-1.22, p=0.16)  1.07 (0.54-2.13, p=0.85) | Ref  2.97 (0.95-9.26 ,p=0.06)  2.02 (0.61-6.66, p=0.25)  2.12 (0.62-7.17, p=0.23) |
| *Index: EPIC-26 – Expanded Prostate cancer Index Composite Short Form, ISUP – International Society of Urological Pathology, PSA - Prostate-Specific Antigen*  ** Statistically Significant* | | | | | |

**Supplementary Table 3** – Unadjusted analysis of prognostic factors for mental wellbeing outcomes in first year of diagnosis

| Outcome | Time Since Diagnosis (Months) | |
| --- | --- | --- |
|  | Unadjusted Analysis  Regression Coefficient (95% CI, p-value) | Adjusted Analysis  Regression Coefficient (95% CI, p-value) |
| Depression (PHQ-9) | -0.04 (-0.09-0.01, p=0.12) | -0.04 (-0.09-0.01, p=0.11) |
| Anxiety (GAD7) | **-0.05 (-0.09- -0.01, p=0.04)*** | **-0.05 (-0.09- -0.01, p=0.04)*** |
| Body Image (BIS) | **0.10 (0.05-0.15, p<0.001)*** | **0.10 (0.05-0.15, p<0.001)*** |
| Fear of Recurrence (FCR7) | -0.03 (-0.12-0.05, p=0.47) | -0.03 (-0.17-0.05, p=0.46) |
| Masculine Self-Esteem (PC-QoL) | **-0.26 (-0.46- -0.06, p=0.01)*** | **-0.26 (-0.46- -0.06, p=0.01)*** |
| *Index: BIS – Body Image Scale, FCR7 – Fear of Cancer Recurrence 7 Scale, GAD7 – Generalised Anxiety Disorder Assessment – 7, PC-QoL - Prostate Cancer Quality of Life Masculine Self-Esteem Subscale, PHQ-9 - Patient Health Questionnaire – 9,*  *** ***Statistically Significant****, Adjusted for age, ethnicity, marital status, co-morbidities, and previous psychiatric history* | | |

**Supplementary Table 4** – Relationship between time since diagnosis and individual mental wellbeing outcomes
